# Emergency Abdominal Aortic Aneurysm Repair in a Patient with Failing Heart: Axillofemoral Bypass Using a Centrifugal Pump Combined with Levosimendan for Inotropic Support

**DOI:** 10.1155/2011/497940

**Published:** 2011-12-18

**Authors:** Pavel Michalek, Pavel Sebesta, Michael Stern

**Affiliations:** ^1^Department of Anesthesiology and Intensive Care, First Faculty of Medicine, Charles University in Prague and General University Hospital, 128 08 Prague, Czech Republic; ^2^Department of Surgery, Central Military Hospital, 169 02 Prague, Czech Republic; ^3^Department of Anesthesiology and Resuscitation, Na Homolce Hospital, 150 21 Prague, Czech Republic

## Abstract

We describe the case of an 83-year-old patient requiring repair of a large symptomatic abdominal aortic aneurysm (AAA). The patient was known to have coronary artery disease (CAD) with symptoms and signs of significant myocardial dysfunction, left-heart failure, and severe aortic insufficiency. The procedure was performed with the help of both mechanical and pharmacological circulatory support. Distal perfusion was provided by an axillofemoral bypass with a centrifugal pump, with dobutamine and levosimendan administered as pharmacological inotropic support. The patient's hemodynamic status was monitored with continuous cardiac output monitoring and transesophageal echocardiography. No serious circulatory complications were recorded during the perioperative and postoperative periods. This paper suggests a potential novel approach to combined circulatory support in patients with heart failure, scheduled for open abdominal aortic aneurysm repair.

## 1. Introduction

Emergency major vascular surgical procedures carry a relatively high mortality risk secondary to factors such as patients' age and associated medical conditions, for example atherosclerosis, hypertension, coronary artery disease (CAD), renal insufficiency, obstructive pulmonary disease (COPD), and diabetes [1–3]. With urgent abdominal aortic aneurysm (AAA) repair, additional factors affect perioperative mortality and serious morbidity—(blood loss, hemodynamic changes related to hypovolemia, aortic cross-clamping and unclamping, and cardiac decompensation) [3, 4]. A variety of strategies for pharmacological and mechanical support of the circulation have been developed for procedures on thoracic and abdominal aorta including catecholamines, temporary axillofemoral bypass, and percutaneous left-heart support [5–7]. This paper describes the use of pharmacological inotropic support utilising the Ca^2+^ receptor sensitizer levosimendan with mechanical support using an axillofemoral bypass and centrifugal pump, under extensive hemodynamic monitoring, in an octogenarian with a failing heart and symptomatic AAA.

## 2. Case Report

An 83-year-old woman was admitted to our department (Type III University Hospital) presenting with abdominal pain located in the umbilical and hypogastric areas. She was conscious (Glasgow Coma Scale 15) and oriented in time, place, and person. Physical examination of the abdomen revealed a pulsatile expanding mass extending downward from the level of the umbilicus. A computed tomography (CT) scan showed a large AAA 10 cm wide, located subrenally.

The patient had a history of CAD, multiple myocardial infarctions, left-heart insufficiency, recurrent attacks of pulmonary edema, and renal insufficiency. Surgical assessment suggested that the aneurysm was not suitable for an endovascular repair due to disturbed aortic anatomy. An epidural catheter was inserted at T10-11 for continuous analgesia. The patient was informed about all risks associated with open procedure and gave written consent.

Echocardiography documented left ventricle dilation with an ejection fraction of approximately 20–25%.

Further, the patient had severe aortic regurgitation, tricuspid regurgitation, medium mitral regurgitation, and pulmonary hypertension (PAP 51/26; mean 39 and PCWP 30 mmHg).

We decided to support distal perfusion and try to attenuate the adverse hemodynamic effects of aortic cross-clamping and its release using an axillofemoral bypass with controlled flow rate using a centrifugal pump.

Cannulation of the right radial and femoral arteries was performed in theatre, and general anesthesia was induced using etomidate, sufentanil, and atracurium. After tracheal intubation, a central venous catheter and pulmonary catheter for continuous oxohemodynamic measurement (Vigilance, Baxter Edwards Labs., Irvine, CA, USA) were inserted via right internal jugular vein.

Baseline parameters documented a critically decreased cardiac index (CI = 1.0 L·min^−1^·m^−2^), with dobutamine administered at a dose of 7 *μ*g·kg^−1^·min^−1^·30 mins later the patient's status continued to deteriorate; she became oliguric, and her systolic blood pressure dropped below 80 mmHg while PCWP rose to 30 mmHg. As intra-aortic balloon pump could not be used, inotropic support with levosimendan (bolus 12 *μ*g·kg^−1^) was initiated followed by continuous infusion at a rate of 0.1 *μ*g·kg^−1^·min^−1^. Right ventricular ejection fraction (Vigilance) increased within 15 mins from 18% to 25%, while the kinetics of left ventricle also improved (TEE) from 20% to 25–30%. CI rose to 1.8 L·min^−1^·m^−2^. A mild decrease in SVR was controlled by the continuous administration of norepinephrine at a dose of 0.02–0.1 *μ*g·kg^−1^·min^−1^. The patient began to pass a small amount of urine (30 mL/hour).

**Figure 1:**
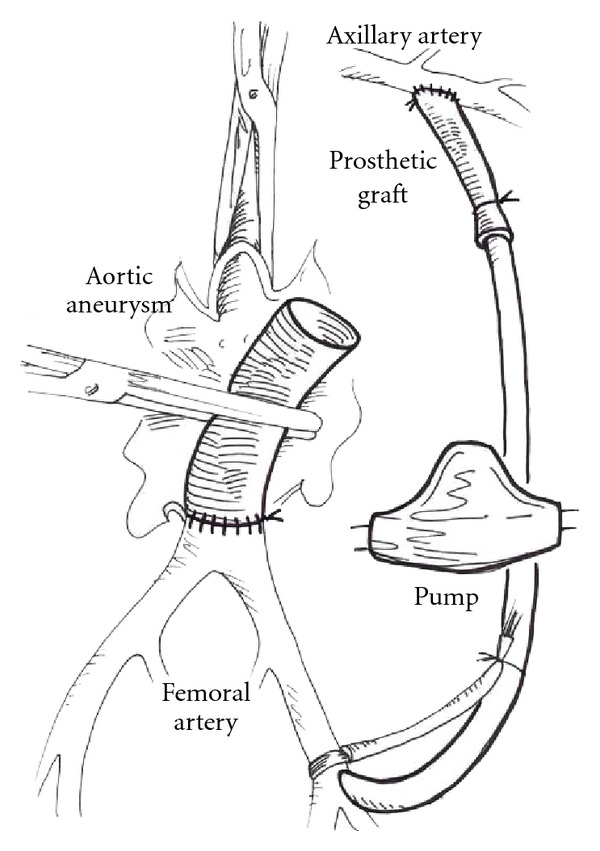
A construction of axillofemoral bypass with a centrifugal pump in the circuit.

The left axillary artery was exposed via a subclavicular incision. Heparin at the dose of 2 mg·kg^−1^ was administered. Due to the cannula/artery diameter mismatch, the appropriate 8 mm PTFE sleeve was end-to-side anastomosed to the axillary artery to host the 28F inflow cannula. Outflow cannula of the same size was driven into the left femoral artery, and the axillofemoral bypass with controlled rate using the centrifugal pump (Jostra, Germany) was introduced (Figure 1). Prior to cross-clamping, the pump flow-rate was set at 1.5 L·min^−1^.

Cross-clamping was associated with minimal hemodynamic response. During the 60 mins of cross-clamping, the pump flow rate was controlled depending on hemodynamic measurements given by pulmonary catheter and TEE. Distal pressure below the clamp was maintained within a range of 45–60 mmHg. Having completed both central and distal aortic anastomoses using 18 mm Dacron prosthesis (Vascutec, Gelseal Co., UK), aortic clamp was gradually released while the bypass flow was decreased and finally stopped. Hemodynamic changes upon declamping were insignificant with an associated lactate level of 2.6 mmol·L^−1^ (baseline 1.6) and no acidosis. Following the standard administration of protamine sulphate and wound suture, the patient was transferred to the vascular ICU. In the early postoperative period, the patient's CI was 2.4–2.7 L·min^−1^·m^−2^, PCWP < 20 mmHg, vital functions were stabilized including diuresis. Inotropic support was progressively reduced (levosimendan left for 48 hrs then replaced by dobutamine). On postoperative day 3, the patient was extubated. The ensuing period was complicated by agitation and chest infection. On postoperative day 6, she was discharged from the ICU and on day 30 dismissed to the nursing home.

## 3. Discussion

The independent predictors for perioperative cardiac events in AAA repair are CAD, history of myocardial infarction, congestive heart failure, and left ventricular dysfunction. The options available to support a failing heart during aortic procedures are limited. Inotropic drugs acting via *β*-adrenergic receptors and PDE-III inhibitors increase the levels of calcium in cardiomyocytes contributing to enhanced arrhythmogenicity and increased myocardial oxygen consumption. Levosimendan acts via Ca^2+^ receptor sensitization, increasing myocardial contractility by improving intracellular calcium utilization. Its proarrhythmogenic potential is minimal, and evidence suggests limited increase in myocardial oxygen consumption [8, 9]. Levosimendan has been extensively studied in medical patients with failing heart and medical or surgical cardiac procedures in patients largely in right-sided heart failure. However, its use in high-risk vascular patients has not been reported.

Mechanical support is another option which can reduce the side effects of aortic cross-clamping and declamping. Clamping results in a decrease in CI, myocardial contractility, and an increase in SVR. The changes are influenced by the site of clamping, degree of collateral perfusion, and preoperative myocardial function [4, 10].

Patients without CAD usually tolerate aortic clamping without major complications. By contrast, a sudden increase in SVR in patients with myocardial dysfunction may be associated with myocardial ischemia [4, 11]. In patients with CAD, autoregulation of coronary blood flow is impaired and increase in intraventricular pressure is not followed by an increase in the subendocardial flow rate. Afterload increase results in acute left-heart insufficiency and a rise in PCWP and LVEDP.

Aortic declamping leads to “declamping shock” which is produced by metabolic and hemodynamic factors. Metabolites from the ischemic tissues below the clamp are released into the systemic circulation. The resultant acidosis further deteriorates the contractility of the ischemic heart [12, 13].

Distal perfusion techniques, used routinely for descending aortic surgery, bring oxygenated blood to tissues below the clamp thus reducing hemodynamic and metabolic changes associated with clamping and declamping [6, 14]. There has been only one case report published describing the use of these techniques in high-risk patients undergoing AAA repair [5]. Lossing et al. performed support of distal perfusion using a previously constructed axillofemoral bypass.

This support did not allow sophisticated regulation of bypass flow. During a sudden drop in SVR or hypovolemia, it is necessary to increase flow in the bypass. These situations mostly occur during sudden blood loss and after declamping.

Placement of a centrifugal pump into the circuit allows controlled support to tissues below the clamp, without the need for full heparinization (ACT 150–500 sec). Construction of an axillofemoral bypass does not significantly prolong the length of procedure (approx. 45 mins). It is appropriate to regulate the pump flow depending on continuous CI measurements, SVR, PCWP, enddiastolic area measurement (TEE), and distal pressure values.

This report is the first case of successful AAA repair in a patient with failing heart and using combined circulatory support with the Ca^2+^ sensitizer levosimendan and controlled distal perfusion with a centrifugal pump. It remains questionable whether levosimendan alone would have provided sufficient hemodynamic support for this patient, but we feel that distal perfusion helped to decrease significantly the sequelae of declamping shock. We are aware that the preferred technique for any high-risk patients presenting with aortic aneurysm would be an endovascular repair (EVAR). However, many aortic aneurysms are not suitable for this endovascular approach because of disturbed aortic anatomy. We suggest that our technique may be beneficial in patients at high cardiac risk contraindicated for EVAR.
